# Supplementary material for: Assessing the effects of agricultural intensification on natural habitats and biodiversity in Southern Amazonia
Source: PLoS One. 2020 Nov 25;15(11):e0225914. doi: 10.1371/journal.pone.0225914 (PMC7688104; doi:10.1371/journal.pone.0225914)
Supplement: Attachment — Submitted filename: Response to Reviewers.docx [file pone.0225914.s002.docx]

- First off, we have to thank the reviewers again for taking the time and risking the nerve while reviewing our manuscript. Your input has considerably helped to improve the manuscript as a whole and the statements made therein.

6. Review Comments to the Author

Please use the space provided to explain your answers to the questions above. You may also include additional comments for the author, including concerns about dual publication, research ethics, or publication ethics. (Please upload your review as an attachment if it exceeds 20,000 characters)

Reviewer #1: I thank the authors for their efforts to address all issues raised. Very appreciated! I do not have additional comments.

- We appreciate the time both reviewers took to thoroughly go through the paper and the extended advice you both gave to make this paper more profound. Responding in kind was the only possible response.

Authors could consider to move text from lines 97-106 before line 88. Authors could check some typos throughout the text, such e.g. at lines 260 ("vertebrate").

- We have moved the corresponding the explanatory text concerning to a more appropriate spot in the introduction **(former l. 97-106, now l. 88-97)**
- We have corrected several typos **(i.e. “vertebarte” to vertebrate, l. 270)**

Reviewer #2: I thank the authors for considering all my comments. Authors made some important changes based on suggestions of both reviewers and I think the manuscript has greatly improved compared to the former version.

However, I still have some minor comments.

i. The inclusion of small “discussion” subsections in the results chapter is a bit strange but I understand this was done to follow comments by Rev1. However, there is no such subsection for chapter 3.1. I suggest changing the name of the small “discussion” subsections with a more informative title (and, hence, avoiding having two subsections with the same name).

- We have changed the titles of the discussion subsections to something more explanatory and hope to increase the informational content now given by the title. **(l. 440-442; 522-524)**
- We did not want to add another subsection in chapter 3.1 because the discussion regarding the content of the respective chapter has been performed thoroughly in another publication. Further, chapter 3.1 is just (as both reviewers have acknowledged correctly) a “summary” of mentioned other publication and is placed in this publication to again point out the background for the subsequent analysis.

ii. The discussion parts require some additional reference to words dealing with future scenarios. Comparisons with similar studies for Amazonia area are needed.

- We have included several further scenario studies for the Amazon region in both discussion subsections. **(l. 448-452; 455-459; 472-474; 550-551; 566-569)**
- Further, some studies (i.e. Galford et al.) used as a “benchmark” in this paper have been conducted as scenario studies but have not been mentioned specifically as “future scenario studies” within this manuscript.

iii. I also think that yours is a “strong” main conclusion at least when reading the abstract! Caution on intensification is, however, highlighted in the conclusion section (I would also stress that results can change between different regions as the two case studies highlighted). These results point out to preferring segregative approaches rather than integrative approaches. I think this (land sparing vs. land sharing and segregative vs. integrative) should be mentioned in the discussion or conclusion section as this work contributes to this “hot topic”.

- Reviewer 2 mentions something we have been discussing in length. We did not want to put the focus on that specific discussion due to several reasons. Although I specifically felt this study to contribute extensively to said discussion. So, I´m happy with having actually been asked to say at least something in contribution to the “land sparing vs. land sharing” debate. **(l. 627-632)**

iv. There are a number of typos and mistakes throughout the text. As examples, L535:..”areas the are domicile to”? L558: See section 1? Did authors mean 3.1? L531: “(86)(5),” in “(5, 86)”?

- The mentioned typos have been corrected. One should expect something like that occurring being at least unlikely after a thorough and professional spelling check. Thanks a lot.
- We deleted the reference to section 1 in l. 558. We actually wanted to refer to that section as it incorporates some negative aspects of employing the BII. After consideration we decided that reference to section 1 isn´t fitting here.

v. L455-460: I suggest dividing this long sentence.

- We have divided the mentioned sentence. **(l. 478-482)**

vi. Why “Deep-Uncertainty” with capital letters? Citation needed?

- Yes, a citation was needed here as we refer to a specific concept framed in a specific publication. Thank you for the hint. **(l. 539)**

vii. Several typos in the Reference list (e.g. L632, L656, L660,..: lack of capital letters in journals’ names.

- The lack of capital letters (journal names) in the reference list have been resolved.
